# Implementation of an intensive short-term dynamic treatment program for patients with treatment-resistant disorders in residential care

**DOI:** 10.1186/1471-244X-14-12

**Published:** 2014-01-18

**Authors:** Ole André Solbakken, Allan Abbass

**Affiliations:** 1 The Department of Psychology, University of Oslo, Forskningsv. 3, Pb. 1094 Blindern, Oslo 0317, Norway; 2 Drammen District Psychiatric Center, Division of Mental Health and Addiction, Vestre Viken Hospital Trust, Drammen, Norway; 3 The Centre for Emotions and Health, Dalhousie University, Hallifax, Canada

## Abstract

**Background:**

This protocol presents a systematic residential treatment- and research program aimed at patients who have not responded adequately to previous treatment attempts. Patients included in the program primarily suffer from anxiety and/or depressive disorders and usually from one or more comorbid personality disorders. The treatment program is time-limited (eight weeks) and has its basis in treatment principles specified in intensive short-term dynamic psychotherapy (ISTDP). This treatment modality is theoretically well-suited for the handling of various forms of treatment resistance presumably central to these patients’ previous non-response to psychological and psychiatric interventions.

**Methods/Design:**

The research component of the project entails a naturalistic longitudinal research design which aims at systematic evaluation of the effectiveness of the program. To our knowledge, this is one of the first treatment programs and corresponding research projects that systematically select patients with previous non- or negative response to treatment and subjects them to a broad and comprehensive, but theoretically unified and consistent treatment system.

**Discussion:**

The present paper introduces the project, describes its theoretical and methodological underpinnings, and discusses possible future implications and contributions of the project. It thereby serves as a comprehensive background reference to future publications from the project.

## Background

It is a well known fact that the majority of patients in psychiatric care respond inadequately to the treatments they are given. This is exemplified in large scale psychotropic medication trials (See STAR-D study). In general, using the notion of effect size [1] most empirically validated psychotherapy treatments tend to help about half of treated patients to a substantial or moderate degree, and about a further quarter of patients to some extent, while the remaining patients usually continue unchanged or are to some extent deteriorated [2]. Using the criteria of clinically significant change [3], the average recovery rate for formal psychotherapeutic treatments in clinical trials (based on meta-analytic studies across a wide variety of psychological treatment models and formats) is between 50 and 60 per cent [2]. Consequently, by this definition, as much as 40 to 50 per cent of patients remain, in terms of clinically meaningful change, unimproved or are worsened at termination. The known deterioration rates from such trials are relatively consistent and indicate that five to ten per cent of patients participating report themselves as reliably worse off after treatment than before [2]. In what is often denoted routine care, i.e. treatment as it is delivered in naturalistic settings without specific supervision of therapists and specific systems for focusing the delivered treatments, the rates of recovery appear to be smaller, so that even more patients are expected to remain unchanged or deteriorate in everyday psychotherapeutic practice [4].

We thus have a situation in which a relatively large proportion of patients in mental health care must be classified as non-responders or negative responders, i.e. they do not benefit from the treatments with which they are provided, or even become worse during treatment. This state of affairs is, or at least should be, distressing to mental health professionals and psychotherapy researchers alike, and any chance of remedying it should be a highly valuable contribution to the mental health care community and the many patients seeking treatment.

There has been very limited research on patients who don’t respond to psychotherapy. It therefore remains largely unknown whether changes in treatment formats, treatment dose, or treatment content could improve outcome for at least a portion of these patients. However, indirect knowledge of this group comes from studies on the treatment of patients with personality disorders [5-8], from studies of non-response to antidepressant medication for depression [9], and from studies of the effects of process-outcome feedback systems on patient responses to treatment [2]. Generally, the findings indicate that the probability of non-response or negative response increases with more severe symptoms, with more profound functional impairment, with more problems in interpersonal relatedness, and with the presence of personality disorder. Problem complexity as evidenced by comorbidity on Axis I and/or II and problem chronicity also appears to predict treatment failure in short-term treatments and has been proposed as an indicator for more complex and broadband treatment [10]. From studies on session by session feedback to therapists and patients about treatment response through monitoring levels of self-reported distress, interpersonal problems, and social role functioning at the beginning of every session, it does, however, appear to be clear that such feedback reduces the prevalence of negative change in psychotherapy, thereby increasing overall effectiveness of treatments [2].

Reviewing the literature, it becomes clear that few studies have selected patients from the group of non-responders to psychotherapy for systematic intervention and scientific investigation. Furthermore, to our knowledge, no studies have *specifically* selected patients primarily based on repeated non-response to treatment and attempted systematic, customized psychotherapeutic intervention with this group. Such studies could yield important knowledge about the possibilities of finding feasible strategies for overcoming this unfortunate state of affairs. If it was possible to identify specific principles of customized treatment that could lead to better outcomes of psychotherapeutic intervention for patients belonging to this group, it would imply significant relief and increase in quality of life for a large number of patients. It could also result in considerable social-economic savings associated with reduced treatment costs and lower reliance on welfare services for a substantial number of those disabled by persistent and unrelenting mental disorders not responsive to traditional treatments.

Accordingly, the current project was designed and implemented. With the aim of relieving the suffering of patients having experienced repeated non-response to treatment efforts, a time-limited residential treatment program was devised. The project was based at the psychodynamic unit at "Thorsberg", the Residential Facility of the Drammen District Psychiatric Center, Norway. The treatment program was primarily directed towards relieving treatment resistant anxiety- and depressive disorders with and/or without comorbid Axis II disorder. This group of patients presumably represents the majority of non-responding or treatment resistant referrals (due to the prevalence of these disorders). As a criterion of inclusion, patients were to have received three or more qualified and documented treatment attempts, defined as separate medical or psychosocial/psychotherapeutic treatment series aimed at the disorder(s) for which they currently were referred for treatment.

Considering the long-standing and chronic disorders suffered by these patients and their often years-long treatment histories, it was clear that the treatment program would have to be organized according to fundamentally different principles than those guiding routine care. First, it was decided that patients were to be treated in residential care in order to increase the probability of treatment compliance (in the medical sense of the word) and reduce the risk of drop-out. Secondly, a clear and non-debatable time limit was provided, with a circumscribed date for terminating treatment for all patients (at the end of eight weeks of residential treatment). Thirdly, a highly intensive treatment and intervention program was devised with multiple treatment components delivered every day. The treatment program was to extensively combine individual psychotherapy, group-psychotherapy, medical/psychopharmacological treatment (if necessary), and various therapeutic activities in groups including body awareness training, art therapy, structured psycho-education, moderate physical exercise, psychosocial training, and milieu therapy. As a theoretical system to inform systematic understanding of patients and all treatment components, the psychotherapeutic model and metapsychology originally described by Davanloo [11] (Intensive Short-term Dynamic Psychotherapy, ISTDP), along with the more general affect theoretical model delineated by affect integration theory [12,13] were chosen. This meant that a fundamental understanding of psychopathology as failed integration of affect, cognition, and behavior was adhered to, with a specified focus on the mobilization of warded off, repressed, or avoided affect associated with pathogenic ruptures to the patient’s bonds with attachment figures throughout the course of development.

Arguably, Davanloo’s ISTDP is the psychotherapy model in the literature today that most clearly conceptualizes systematic work with treatment resistance, at least in the traditional psychodynamic sense of that word. ISTDP posits a conceptually integrated and extensive set of interventions and intervention modes that are specifically directed at dealing with those maneuvers patients often consciously or unconsciously resort to in order to avoid genuine emotional closeness, to water down strong affect, to remain passive, compliant, or defiant and so on and so forth. These processes presumably contribute substantially to the generation of obstacles to treatment and consequent treatment failure if not effectively and specifically dealt with. For this reason we chose to use the technical intervention apparatus described and detailed by Davanloo and others (e.g. [14,15]) as a basis for the individual psychotherapy courses, and to use adjusted and adapted versions of this system for guiding intervention in other components of the treatment.

In general, ISTDP is a fairly well documented treatment for a number of mental disorders. It has been demonstrated to be clinically effective and cost-effective in case series of mixed psychiatric samples [16,17]. Also, it has been shown to be effective and cost effective with a previous (but small) sample of patients with treatment resistant depression [18], in personality disorder [5,19-21], and in a specialized hospital setting for treating severe cases of borderline personality disorder, most of which had had previous treatment but failed to benefit [22]. It has been studied in resistant and complex populations in several case series and randomized controlled trials [19]. It has been demonstrated in the National Health Services in the UK’s "Pathfinder" project to produce good treatment effects in patients resistant to other treatment efforts [23]. It has been found effective for patients with chronic somatic conditions with functional movement disorders [24], with chronic pain [25] and with medically unexplained symptoms with repeated emergency visits [26]. Thus, there is a fairly good empirical basis for choosing the ISTDP model as a basis for a treatment program aimed at relieving the suffering of patients characterized by non-response to previous treatment attempts.

The treatment philosophy and treatment principles of ISTDP have been described in detail elsewhere, e.g. [5,11,15,22]. Here it suffices to say that the main emphasis of ISTDP is to rapidly help the patient acknowledge, identify, experience, and express unconscious and warded-off affects or emotions that theoretically are postulated to produce unconscious anxiety, symptom disturbances, and various defensive strategies that become interpersonally maladaptive and may produce characterological disturbances within the patient. The main technical interventions are to detect, clarify and challenge affect-avoiding strategies or defenses in systematic collaboration with the patient, while inviting and encouraging clear awareness and deep experience of warded-off feelings. This process, when effectively executed, mobilizes what Davanloo called "complex transference feelings" with the therapist, thereby actualizing the patient’s most pressing patterns of attachment-related affect and his most characteristic coping strategies with these affects within the therapeutic relationship. Simultaneously, the conscious and unconscious therapeutic alliance is mobilized, producing a force within the patient that allies with the therapist in the face of avoidance strategies and resistances [11]. In turn, this leads to a reduction in defensive coping so that the patient can more directly work through unresolved feelings related to broken attachments in the past and subsequent trauma [5].

The treatment program and its quality control system were designed so that comprehensive and reliable data would be available for determining the ultimate effectiveness of the treatments delivered. Such documentation was seen to be paramount in order to justify the relatively large investments of resources and money that is necessary for offering residential care for patients that statistically would be likely to fail also this treatment. A substantial number of process- and outcome indicators were therefore implemented as standard procedure. This quality control system implied that all individual treatment sessions were to be videotaped, the systematic use of diagnostic and functional interviews prior to, at termination and follow-up after termination, and that an extensive battery of self-report measures were to be administered prior to treatment, during treatment, and after treatment. Furthermore, a system for session-by-session process and progress tracking was to be implemented, so that therapists would have continuous feedback about the development of all patients. The follow-up period post treatment termination was defined to be at least 12 months. The research relevance of this data was immediately acknowledged; therefore a research protocol was detailed that was to hold international standards in terms of assessment procedures and methodological design.

The data gathered through the "Drammen Project" are unique in that they combine the selection of a group of patients previously not systematically investigated in the established literature, while at the same time delivering a multifaceted treatment program of unusual intensity based on a treatment model that appears especially well-suited for the task of relieving treatment resistance. These data thus carry potentially important insights that may be highly valuable to both psychotherapy research and the psychotherapy community.

### Main research objectives for the Drammen project

The following research objectives can be detailed as being at the heart of the research part of the Drammen Project for treating patients with treatment resistant mental disorders.

1. What is the effectiveness of an intensive multifaceted time limited treatment program based on principles from ISTDP for patients with known treatment resistant disorders at termination and follow-up after treatment?

2. Which process-outcome mechanisms may foster or conversely hinder adequate treatment response for patients entering the program?

3. Which patient- and therapist factors predict poor and good response for patients entering the program?

## Methods/Design

### Treatment setting and location

Treatment is provided at the Psychodynamic Unit at "Thorsberg", the Residential Facility at the Drammen District Psychiatric Center, Norway. This unit was developed and founded with the task of managing difficult-to-treat conditions through intensive, time-limited in-patient care. The unit has room for a total of six patients, each receiving an eight week "block" treatment program. Thus, groups of up to six patients enter and terminate treatment simultaneously. Each year a total of five groups or "blocks" are treated with a maximum total of 30 patients per annum.

### Recruitment procedures

Patients are recruited among referrals for in-patient psychiatric care at the residential treatment facility of the Drammen District Psychiatric Center. Patients are referred partly by practitioners at local out-patient psychiatric clinics, nearby psychiatric hospitals, and by general practitioners in the Drammen area. Patients are initially screened for the inclusion/exclusion criteria by the intake-team at the residential facility, before completing an evaluation session with one of the treatment providers at the psychodynamic ward. The evaluator reaches a final decision regarding inclusion/exclusion on the bases of prior treatment history, existing diagnostic information, referral information, and the patients’ responses to intervention in the evaluation session. Patients who are offered treatment at the ward are then informed about the study and invited to participate. If patients are willing and eligible to participate, signed and informed consent is obtained after comprehensive explanation of the procedures of the project. Participants do not receive compensation in any form for evaluation, screening, or assessments. Participating in the assessment-procedures is obligatory as part of internal quality control for patients receiving the treatment program, however participation in research project is voluntary.

### Participating patients

#### Inclusion criteria

Adult patients (aged 18–70) are eligible to participate if they a.) Fulfill basic criteria of need for hospitalization for psychiatric treatment including insufficient general functioning and loss of mastery/function in multiple domains, e.g. inadequate self-care, substantial breakdown in relational, occupational, and/or personal functioning. b.) There are clear indications of prior treatment resistance, i.e. previous failure to respond adequately (in terms of significant symptomatic relief and increased functioning) in three or more prior treatment attempts for the current episode/ongoing psychiatric disease. The number of three previous attempts were chosen to increase the likelihood that included patients had severe and truly treatment resistant disorders, rather than previous non-response being attributable to chance factors, or the delivery of poor or unsuitable treatment. And c.) The patient demonstrates signs of capacity for taking an intrapsychic perspective on own problems during the evaluation session, i.e. the ability to regard one’s problems as the result of difficulties in dealing with feelings, thoughts, and reactions to self/others. All three criteria are to be fulfilled for inclusion. Co morbid axis-I and axis-II disorders are allowed, as is medication with the exception of continuous intake of sedatives.

#### Exclusion criteria

Patients are excluded from the study if they suffer from one or more of the following disorders; a psychotic disorder (except short, reactive psychotic episodes), bipolar disorder type I, dissociative identity disorder, addiction of such severity that detoxification is indicated (after which entering treatment is possible), psychiatric disorders secondary to medical conditions, mental retardation, or insufficient command of the Norwegian language hindering the utilization of treatment. Also, acute suicide risk and history of severe acting out/problems with impulse control are considered contraindications to participation.

### Therapists

All therapists providing individual treatment in the program are trained and certified psychologists. With the exception of one psychologist who left the unit soon after the program was started, all therapists participate in a three-year core training in ISTDP (Intensive Short-Term Dynamic Psychotherapy) including supervision of video-taped therapy sessions. The core training program is delivered in Norway by internationally renowned tutors organized and directed by Jon Frederickson, Washington School of Psychiatry, US. In addition to Frederickson the team of tutors consists of Kees Cornelissen, PTC De Viersprong, Netherlands, and Professor Allan Abbass, Dalhousie University, Canada. In addition, the therapists take part in bimonthly group supervisions with Professor Allan Abbass. Finally, peer supervision takes place on a weekly basis in the unit. The level of experience of individual therapists is highly variable ranging from 2 to over 30 years.

The group-psychotherapists are highly experienced, trained, and certified in traditional psychodynamic group psychotherapy and have co-developed the synthesis of ISTDP-principles and traditional group therapeutic ideas adhered to in the program. The body awareness instructor/physiotherapist is highly experienced, specialized and certified in psychomotor physiotherapy. Finally, the therapist administering art therapy is certified and highly experienced in this modality.

### The treatment program

The treatment program is an eight week residential intervention, within these eight weeks a number of treatment components are delivered; the majority of which are actively integrated within the theoretical frameworks of ISTDP [11] and affect integration theory [12]. Patients enter the program in groups of up to six patients (minimum four), each member of a group enters treatment at the same time and terminates eight weeks later. Patients receive either two weekly individual single sessions (45 minutes) or one weekly double session (90 minutes) depending on their capacity for tolerating intensive, relatively long-lasting pressure and focus toward the experiencing of warded off feelings.

All individual therapists were educated and trained in the basic principles of ISTDP, and instructed to adhere to these principles in individual sessions. Treatment integrity and adherence to ISTDP-principles are continuously checked in the bimonthly group supervisions with Professor Allan Abbass. Also, videotapes of all individual sessions are available for systematic adherence and competence evaluation.

Patients also receive two weekly group sessions 90 minutes each. These group sessions integrate the notion of pressure to feeling, and systematic clarification and challenge of defenses, with traditional group therapeutic principles [27]. There are also weekly body awareness training groups based on principles from psychomotor physiotherapy, along with bi-weekly low-intensity physical exercise (walking), and weekly psycho-educational lectures providing a conceptual understanding of the therapeutic process. Finally, all patients take part in weekly art therapy groups focusing on the experience and expression of feelings through creative and artistic displays. Individual psychomotor therapy is an optional addition offered to all patients at the start of treatment.

Beyond the individual therapist, all patients are provided a primary treatment contact among the ward staff with whom they are encouraged to discuss important aspects of their development and potential challenges for the therapeutic process in a systematic and continuous fashion. The primary contact and other staff also implement treatment principles from ISTDP whenever suitable and indicated in the every day on-goings within the ward. Table 1 summarizes the weekly treatment activities of the program.

**Table 1:** Weekly treatment activities for patients included in the program

**Activity**	**Duration (minutes)**	**Weekly sessions**
Individual psychotherapy*	50	2
Group psychotherapy*	90	2
Body awareness training*	60	1
Art therapy*	90	1
Physical exercise	120	2
Lectures*	60	1
Day planning in plenum*	30	5
Consultation with miljeu-therapist/primary contact*	45	1
Group based social activity/training	120	3
Psychomotor physiotherapy (optional)*	45	(1)
Consultation with social/occupational therapist (optional)	45	(1)
Arts and crafts workshop (optional)	120	(1)

Note. *Specifically tailored be in keeping with ISTDP-treatment principles.

### Scientific design

The research project is a naturalistic longitudinal study for examining the short- and long term effectiveness of this time-limited intensive in-patient treatment program for relieving treatment resistant conditions, mainly anxiety-, depressive-, and personality disorders. To increase confidence in our findings a naturalistic waitlist control condition is established. Thus, patients are assessed with self-rated measures prior to their evaluation session so that development from the time of evaluation until the onset of treatment can be used as a contrast to effects presumably attributable to the treatment itself. In the treatment and follow-up phases, observer- and self-rated assessments are done in the week prior to hospitalization (T1), during the eight week treatment phase (t01-t08), at termination (T2), in addition to six (T3) and twelve months (T4) after the end of treatment. Individual psychotherapy sessions are videotaped for supervision purposes and made amenable to the application of observational coding systems for rating various process and outcome factors in treatment.

### Assessments and assessment schedule

#### Observer rated measures

Experienced members of staff are specifically trained for assessing patients included in the treatment program on the relevant observer based measures which are conducted prior to hospitalization (T1), at termination (T2), and twelve months after termination (T3). The MINI Neuropsychiatric Interview [28] is used for assessing Axis I diagnoses. The Structured Clinical Interview for the Diagnostic and Statistical Manual of Mental Disorders, Axis II, Fourth Edition (DSM-IV-R) (SCID-II) [29] is used for assessing Axis II personality disorders and severity of personality problems. Finally, the Affect Consciousness Interview (ACI) [30] is used for assessing the functional level of affect integration and affect organization.

##### 

**The MINI neuropsychiatric interview** The Mini International Neuropsychiatric Interview [28] was developed to meet the need for a brief, reliable and valid structured diagnostic interview for screening a variety of psychiatry disorders and replacing the somewhat cumbersome Structured Clinical Interview for DSM-IV-R, Axis I (SCID I) and the Composite International Diagnostic Interview (CIDI) in clinical trials. The MINI contains 120 questions and screens 16 Axis I and one Axis II DSM IV-R disorders for 24 current and lifetime diagnoses. Like the SCID-P and CIDI, the MINI is organized in diagnostic sections. Using branching tree logic, the MINI has two to four screening questions per disorder. Additional symptom questions within each disorder section are asked only if the screen questions are positively endorsed. Since anxiety and depressive disorders are a central focus of the program, the presence or absence of such disorders will be used as specific measures of outcome from the treatment, along with diagnostic status on other relevant diagnostic categories in the MINI.

##### 

**The structured clinical interview for the diagnostic and statistical manual of mental disorders, axis II (SCID-II)** The SCID II [29] is a version of the SCID developed for the assessment of DSM-IV-TR Personality Disorders. The interview covers the eleven DSM-IV Personality Disorders (including Personality Disorder NOS) and the appendix categories Depressive Personality Disorder and Passive-Aggressive Personality Disorder. It closely follows the language of the DSM-IV Axis II Personality Disorders criteria. The scoring is done so that the trait either is absent, sub-threshold, true, or there is "inadequate information to code". Traits considered true are then summed up and diagnoses are indicated when the required number of traits are present for any given disorder. The SCID-II is administered by trained interviewers and generally yields decent indications of the personality disorder spectrum.

##### 

**The Affect Consciousness Interview (ACI)** The ACI is a semi-structured interview designed for assessing the consciousness and integration of discrete affects (Affect Consciousness - AC) [30,31]. Five aspects of the experience of these affects are assessed: Scenes/elicitors, awareness, tolerance, emotional (nonverbal) expression and conceptual (verbal) expression. The interviewer asks about the following for each affect: (1) scenes in which the affect is activated, (2) how the patient becomes aware of and recognizes the affect, (3) how the affect impacts upon the patient, how the patient copes with the affect, and what information the patient decodes from the affect activation, (4) to what extent and how the affect is expressed in nonverbal forms (i.e., emotional expression, a category that refers to the respondent’s consciousness of own voluntary and involuntary nonverbal expressions) and finally (5) to what extent and how the affect is expressed verbally (i.e., conceptual expression). The interviews are administered by trained interviewers, videotaped, and scored according to criteria specified in the Affect Consciousness Scales (ACSs) [30]. The ACSs comprise four specified nine-step scales, one for each of the integrative aspects: Awareness, Tolerance, Emotional Expression and Conceptual Expression. A score of 1 is the lowest possible, 9 is the highest, and a score of 5 would be considered normal. On the basis of these indicators, scores on three different levels are calculated: Overall mean score (Global AC), mean score on each of the four integrating aspects (e.g., the mean Awareness-score across the nine affects), and mean score on each of the specific affects (e.g., mean score on awareness, tolerance, emotional expression and conceptual expression for Tenderness/Care).

#### Self rated measures

A number of self-rated questionnaires are completed prior to the evaluation session, prior to treatment onset, and throughout the treatment- and follow-up phases, reflecting various aspects of patient functioning and therapeutic process. One questionnaire assesses basic demographic information, along with information on prior treatment, educational level, current occupational status, the use of medications, and current financial situation. As a principle measure of level of psychopathology and psychological functioning, as well as a process measure reflecting weekly improvement/deterioration the Outcome Questionnaire- 45 (OQ-45) is used [32]. The OQ-45 is first administered before the evaluation session, in the week before onset of treatment, and then before every single individual psychotherapy session throughout the treatment phase, as well as six and twelve months after termination. In addition, the Symptom Checklist 90-Revised [33] (SCL-90-R), and the Inventory of Interpersonal Problems (IIP-64) [34] are used prior to evaluation, before onset of treatment, in weeks two and five of treatment, at termination, and six and twelve months after termination. The Working Alliance Inventory, patient version (WAI-P) [35] is used as a measure of conscious working alliance and is administered in week two, five, and at termination.

##### 

**Outcome questionnaire- 45 (OQ-45)** The OQ-45 is a symptom and distress inventory developed by Lambert et al. [32]. It has been found to be useful for examining the effectiveness of psychotherapy over time [36]. The instrument is designed to assess "patient functioning" and scores are used to track changes in symptomatology on a session-by-session basis. The OQ-45 consists of 45 items tapping various aspects of psychological distress each associated with a 5-point Likert scale. Responses refer to the last seven days and range from "never" to "almost always". Once responses to nine negatively worded items have been reverse-coded, sum scores are calculated with higher scores representing increasing levels of psychopathology. Overall scores are calculated along with scores on each of three subscales, assessing Symptom Distress (SD), Social-Role functioning (SR) and Interpersonal Relationships (IR) respectively.

##### 

**Symptom checklist-90-revised (SCL-90-R)** The SCL-90-R is a widely used and comprehensive symptom inventory that measures symptom distress on nine dimensions and three global indexes [33]. Intensity of 90 symptoms during the last seven days is rated on a five point Likert scale ranging from not at all (0) to very much (4). The Global Severity Index (GSI), the average score across all 90 items, is regarded a highly nuanced and valuable indicator of overall current level of distress [37]. The primary symptom dimensions that are assessed are somatization, obsessive-compulsive, interpersonal sensitivity, depression, anxiety, hostility, phobic anxiety, paranoid ideation, psychoticism, and a category of "additional items" assessing other aspects of patient symptoms such as sleep disturbances, guilt/self-blame, and eating/appetite problems. The depression-, anxiety-, and phobic anxiety- subscales will be used as specific outcome measures of these, to the program, central outcome dimensions.

##### 

**Inventory of interpersonal problems (IIP-64)** General and specific interpersonal problems are assessed using the 64 item IIP-circumplex version [34]. The IIP-64 consists of two types of items. The first 39 items begin with the phrase: "It is hard for me to…" The remaining 25 items represent "Things that you do too much." Each item is rated on a five point Likert scale ranging from not at all (0) to very much (4). The general or elevation factor of the IIP-64 has been consistently linked to both symptom severity and negative affectivity [38]. The second (Agency) and third (Communion) factors, yielding the IIP-64 circumplex structure, generally show good construct validity in terms of fit with a quasi-circumplex model, along with distinct convergent-discriminant correlation patterns with different forms of personality pathology, supporting the notion that the IIP-64 model adequately represents its theoretically alleged distinctions in interpersonal functioning [31,39]. The overall score of the IIP-64 is used as an indicator of general interpersonal problems. The IIP-64 also yields eight octant sum-scores indicating specific problems with being PA = Domineering/Controlling, BC = Vindictive/Self-Centered, DE = Cold/Distant, FG = Socially Inhibited, HI = Non-Assertive, JK = Overly Accommodating, LM = Self-Sacrificing and NO = Intrusive/Needy.

##### 

**The working alliance inventory (WAI)** Patient rated working alliance is assessed by the Working Alliance Inventory (WAI) [31] short version with 12 items rated on a seven point scale [40]. The WAI covers three aspects of working alliance: therapeutic bond, task, and goal. The goals subscale assesses the extent to which patient and therapist agree on the goals that are the target of the intervention. The tasks subscale assesses the extent to which patient and therapist agree on the in-counseling behaviors and ideas that form the substance of the counseling process. The bond subscale assesses the extent to which patient and therapist share mutual trust, acceptance, and confidence in the process at hand. Table 2 summarizes the assessment instruments and assessment schedule.

**Table 2:** Overview of assessment instruments and assessment schedule in the treatment and follow-up phases

	**T1**	**T01-08**	**T2**	**T3**	**T4**
***Clinical and diagnostic interviews***					
MINI 5.0, Axis I diagnoses (symptom disorders)	**X**		**X**		**X**
SCID-II, Axis II diagnoses (personality disorders)	**X**		**X**		**X**
The affect consciousness interview (ACI)	**X**		**X**		**X**
***Background information***					
Historical background and previous treatments	**X**			**X**	**X**
Use of medications	**X**		**X**	**X**	**X**
Work, education, and financial situation	**X**			**X**	**X**
***Symptoms***					
Symptom check List 90- R, SCL-90-R	**X**	**X**	**X**	**X**	**X**
Outcome questionnaire- 45, OQ-45	**X**	**X**	**X**	**X**	**X**
***Interpersonal functioning***					
The inventory of interpersonal problems-64 (IIP-64)	**X**	**X**	**X**	**X**	**X**
***Therapeutic alliance***					
Working alliance inventory-short patient (WAI-S-P)	**X**	**X**	**X**		

Note. T1 = pre treatment, T01-08 = sessions 1 through eight during treatment, T2 = termination, T3 = 6 months post treatment, T4 = 12 months post treatment.

### Sample size and data analytic strategies

#### Sample size

The research project is designed to include a minimum of 250 patients; however, all patients treated at the unit are invited to participate, so the sample size is expected to increase further over time.

#### Statistical power

In research where demonstrating the effectiveness of treatment is an objective, statistical power will always be an important factor. Exact estimation of statistical power is only possible for specific analytic designs aimed at delimited research questions and necessarily based on conditions that are not exhaustively known when a study is initiated. However, in general a sample of 250 patients will yield a statistical power of .80 for the detection of small to medium effect sizes for the overall effect in one-way analyses of variance. This means that the probability of detecting significant differences between points of measurement is 80% for an effect size (Cohens’s d) greater than 0.3 -0.5. This estimate will also apply to change scores between to points of measurement if we assume that the average correlation between time-points is greater than .50. In pair-wise comparison of subgroups with n = 80 the probability of detecting small to medium effects similarly will be about .80.

#### Data analytic strategies

The data from the project lends itself to various research topics. Most centrally, the data can contribute valuable knowledge on the effectiveness of short-term and time-limited residential treatment for treatment resistant psychiatric conditions. For answering this research question using continuous outcome variables (e.g. OQ-45, IIP-64, AC) multilevel (or hierarchical linear) modeling will be applied using the linear mixed models module in the SPSS/PASW, version 18.0. The application of multilevel modeling for the analysis of longitudinal data, in this case repeated measurements on the same individuals, is strongly recommended in the literature [41,42]. For longitudinal data, measurements are nested within individuals, so that measurements represent units at the first level and individuals represent units at the second. It is usually proposed that as requirements for longitudinal analyses, all variables must be collected at three or more measurement waves, that a continuous outcome changes systematically over time, and that a meaningful unit for time is included [42]. Each of these requirements is met by the design of the present project. Generally, based on experiences with previous patients going through the program we expect low attrition rates. Almost all patients are expected to complete the treatment program and deliver data for the complete treatment phase. Some attrition is expectable for the follow-phase, but here to, based on previous experience, we expect that as much as 80 – 90% will deliver follow-up data at least on one occasion.

Using Cohen’s [1] formula, effect sizes can be calculated as the growth-curve estimated difference between end-status and baseline divided by the pooled standard deviation across all measurement points of the relevant outcome variable. For dichotomous outcome variables (e.g. presence or absence of Axis I and II disorders) treatment effects will be tested with simpler survival analysis and cross tabulation.

Treatment response for individual patients on self-rated instruments is defined in terms of clinically significant change (CSC) according to Jacobson and Truax [3]. Thus, we will identify the number of patients that are reliably improved and have moved from the dysfunctional to the functional range (recovered), the number of patients reliably improved but not recovered, the number of patients reliably worsened, and the number of patients unchanged on central outcome measures.

The data also lend themselves to a number of investigations into the conceptual and empirical relationships between various measures of psychological health and functioning, and to tests of the psychometric properties of different measures included in the study, e.g. on the relationship between symptom patterns and interpersonal problem types, on the validity of the subscales of the OQ-45 and the SCL-90-R and so on and so forth. For such investigations traditional exploratory and semi-confirmatory factor analyses, multiple linear regression models, and correlation analyses will be performed. Finally, data may prove to be a valuable source of information on the predictors of change and treatment response when treating treatment resistant patients. For answering research questions related to this topic, individual growth curves will be utilized and relevant predictors entered into the multilevel models of change.

### Research ethical considerations

Participation in the research part of the program is voluntary. Participants are informed that they can withdraw their consent to participate at any time without disclosing reasons for their cancellation. There are no negative consequences of such withdrawal for the treatment delivered. All participants sign a written and informed consent. The protocol for the study was evaluated by the Regional Committee for Medical and Health Research Ethics in Eastern Norway and a letter of exemption was issued classifying the study as a quality control project approving dissemination of results.

## Discussion

The Drammen Project has as its core a systematic treatment program directed at patients who have not profited adequately from previous treatment attempts. Included patients primarily suffer from anxiety or depressive disorders, and usually, but not always, from comorbid personality disorders. The treatment program is time-limited and based in treatment principles from intensive short-term dynamic psychotherapy, a treatment modality that theoretically is well-suited for the handling of various forms of treatment resistance presumably central to these patients’ previous non- or negative response to therapy. The project furthermore entails a naturalistic longitudinal research design which systematically evaluates the effectiveness of the treatment program. To our knowledge, this is the first treatment program and corresponding research project that systematically selects patients with previous non- or negative response to treatment and subjects them to a broad and comprehensive, but theoretically unified and consistent treatment system. The project thus carries the potential of yielding important and novel knowledge about the treatment of non-responders to psychiatric care and the potential for succeeding with them through intensive and systematic intervention. Furthermore, the program is one of very few that applies principles from ISTDP across a variety of treatment components within a residential setting. Results from the Drammen Project will thus be a valuable complement to the promising findings already reported on residential ISTDP [22].

The protocol presented here gives an in depth description of the treatment program and the corresponding research design. It offers a comprehensive source of background information pertinent to the Drammen Project and any publications that will arise from it. It thus will serve as a central reference that contains more detailed information on the methodological and design qualities of the project than is possible in later research publications and make these qualities available for critical review.

### Strengths and limitations

The Drammen Project does meet many requirements for a sound observational longitudinal treatment study. The number and quality of assessment instruments are generally high and there is a systematic combination of observational and self-rated measures. Assessments are done at a large number of occasions, increasing the reliability of measurements. The data collected are well suited for hierarchical or multilevel modeling approaches [42], making modern and powerful data analytic strategies for longitudinal data feasible. Patients are assessed not only prior to, during and at treatment termination, but also during a follow-up period of reasonable duration. Furthermore, the group of patients selected for the project has not previously been studied systematically, but represent an important sub-population of treatment takers that suffer substantially despite seeking and receiving treatment. The project also will generate data for a fairly large number of patients and will comprise a relatively large number of therapists. As the treatments are implemented in a naturalistic setting within the Norwegian public health care system we have reason to believe that results should generalize to other naturalistic treatment settings, i.e. other residential treatment institutions if similar systems were to be implemented there.

One limitation of the project is its inability to differentiate the effects of various sub-components of the treatment program. It is thus impossible to discern whether effects are generated primarily by certain components of the program (individual treatment, group therapy etc.), or by the synergy of all components. Likewise, it is impossible to discern the effects of theory specific therapeutic factors, such as those entailed by the ISTDP- treatment framework, and the effects of general or common therapeutic factors such as a safe environment for self-disclosure, warmth, trust, respect, corrective emotional experience, exposure, support, psycho-education etc. However, the effectiveness of the entire treatment system will be demonstrable.

There is no active control condition, nor randomization of subjects, so it is not possible to show the efficacy of this treatment compared to other relevant treatment systems or to treatment as usual (TAU). Such studies would constitute a valuable and important continuation of the work started here. However, the selection of patients on the basis of previous treatment failure does imply an inherent form of control condition. In previous qualified attempts of treatment these patients have failed to respond adequately, thus significant effects detected in the present program will indicate at least indirectly the efficacy of the program as compared to treatment as usual. Also, the use of waiting list data as a naturalistic control condition increases the credibility of findings from the project.

### Possible contributions to the field of knowledge

There are primarily two valuable contributions to the literature that may result from the Drammen Project. Firstly, it can demonstrate the effectiveness of specialized intervention for patients suffering from treatment resistant disorders. If the Drammen projects and its intervention system prove to be effective, this will have implications for treatment providers in mental health care across many nations and health care systems. Furthermore, the demonstration of such effects could form the basis for new studies aimed at identifying mechanisms of change for this group on a larger scale. The Drammen Project also will generate substantial data which on their own may shed light on the question of process-outcome relationships in the treatment of treatment resistant disorders. Likewise, patient and therapist predictors of treatment response in this group can be identified, along with significant information on the pre-treatment characteristics of treatment resistant patients in general.

Secondly, the Drammen Project may demonstrate the utility of a broad scoped intervention system based on ISTDP in a residential setting. Prior investigations into the effectiveness of ISTDP and ISTDP-based treatments have been conducted in out-patient settings situated outside of public health care systems, with the exception of the work done by Cornelissen and his collegues in the Netherlands [22]. Thus, the present project can complement previous research by adding knowledge about residential ISTDP with a different patient population and implemented in another residential facility in another public health care system than has been the case in previous research.

## Competing interests

To the best of the authors’ knowledge there are no competing interests, neither financial nor non-financial, that could influence the results of any studies within the present project.

## Authors’ contributions

OAS planned and implemented the project, contributed as one of the study therapists at the site, and drafted the manuscript. AA participated in the design of the study, served as supervisor for the study therapists and helped in drafting the manuscript. Both authors read and approved the final manuscript.

## Pre-publication history

The pre-publication history for this paper can be accessed here:

http://www.biomedcentral.com/1471-244X/14/12/prepub

## References

[B1] Cohen J Statistical Power Analysis for the Behavioral Sciences 1988 2 Hillsdale, NJ: Lawrence Erlbaum Associates

[B2] Lambert MJ Ogles BM Lambert MJ The efficacy and effectiveness of psychotherapy Bergin and Garfield’s handbook of psychotherapy and behavior change 2004 5 New York: Wiley 139 193

[B3] Jacobson NS Truax P Clinical significance: a statistical approach to defining meaningful change in psychotherapy research J Consult Clin Psychol 1991 59 12 19 2002127 10.1037//0022-006x.59.1.12

[B4] Wampold BE The great psychotherapy debate: models, methods, and findings 2001 Mahwah, NJ: Lawrence Erlbaum Associates

[B5] Abbass A Sheldon A Gyra J Kalpin A Intensive short-term dynamic psychotherapy for DSM–IV personality disorder: a randomized controlled trial J Nerv Ment Dis 2008 196 211 216 10.1097/NMD.0b013e3181662ff0 18340256

[B6] Monsen JT Odland T Faugli A Daae E Eilertsen DE Personality disorders: changes and stability after intensive psychotherapy focusing on affect consciousness Psychother Res 1995 1995 5 33 48

[B7] Giesen-Bloo J van Dyck R Spinhoven P van Tilburg W Dirksen C van Asselt T Kremers I Nadort M Arntz A Outpatient psychotherapy for borderline personality disorder-randomized trial of schema-focused therapy vs. transference focused therapy Arch Gen Psychiatry 2006 63 649 658 10.1001/archpsyc.63.6.649 16754838

[B8] Town J Abbass A Hardy G Short-term psychodynamic psychotherapy for personality disorders: a critical review of randomized controlled trials J Pers Disord 2011 25 723 740 10.1521/pedi.2011.25.6.723 22217220

[B9] Trivedi MH Fava M Wisniewski SR Thase ME Quitkin F Warden D Ritz L Nierenberg AA Lebowitz BD Biggs MM Luther JF Shores-Wilson K Rush AJ Medication augmentation after the failure of SSRIs for depression N Engl J Med 2006 354 1243 1252 10.1056/NEJMoa052964 16554526

[B10] Clarkin JF Levy KN Lambert MJ The influence of client variables on psychotherapy Bergin and Garfield’s handbook of psychotherapy and behavior change 2004 5 New York: Wiley 139 193

[B11] Davanloo H Unlocking the Unconscious 1990 Chichester: Wiley

[B12] Solbakken OA Hansen RS Monsen JT Affect integration and reflective function: clarification of central conceptual issues Psychother Res 2011 21 482 496 10.1080/10503307.2011.583696 21623546

[B13] Monsen JT Monsen K Goldberg A Affects and affect consciousness: A psychotherapy model integrating Silvan Tomkins’ affect- and script theory within the framework of self-psychology Pluralism in self-psychology: Progress in self-psychology, Vol. 15 1999 Hillsdale, NJ: Analytic Press

[B14] Frederickson J Co-creating Change: Effective Dynamic Therapy Techniques 2013 Seven Leaves: Kansas City

[B15] Coughlin Della Selva P ten Have-de Labije J Dynamic assessment of ego functioning in Davanloo’s ISTDP The Working Alliance in Intensive Short term Dynamic Psychotherapy 2001 The Netherlands: The Netherlands Foundation for Intensive Short-term Dynamic Psychotherapy

[B16] Abbass A Intensive short-term dynamic psychotherapy in a private psychiatric office: clinical and cost effectiveness Am J Psychother 2002 56 225 232 12125299 10.1176/appi.psychotherapy.2002.56.2.225

[B17] Abbass A The cost-effectiveness of short-term dynamic psychotherapy J Pharmacoecon Outcomes Res 2003 3 535 539 10.1586/14737167.3.5.535 19807387

[B18] Abbass A Intensive short-term dynamic psychotherapy for treatment resistant depression: a pilot study Depress Anxiety 2006 23 449 552 10.1002/da.20203 16845654

[B19] Abbass A Town J Driessen E Intensive short-term dynamic psychotherapy: a systematic review and meta-analysis of outcome research Harv Rev Psychiatry 2012 20 97 108 10.3109/10673229.2012.677347 22512743

[B20] Hellerstein DJ Rosenthal RN Pinsker H Samstag LW Muran JC Winston A A randomized prospective study comparing supportive and dynamic therapies: outcome and alliance J Psychother Pract Res 1998 7 261 271 9752637 PMC3330512

[B21] Winston A Laikin M Pollack J Samstag LW McCullough L Muran JC Short-term psychotherapy of personality disorders Am J Psychiatry 1994 151 190 194 8296887 10.1176/ajp.151.2.190

[B22] Cornelissen K Verheul R Treatment outcome of residential treatment with ISTDP AD HOC Bull Short Term Dynamic Psychotherapy 2002 6 14 23

[B23] Hajkowski S Buller S Short-Term Psychodynamic Psychotherapy (STPP): Preliminary Results from a Naturalistic Process and Outcome Study of Treatment of Severe, Complex and Treatment Resistant Disorders 2013 Oxford: Annual Meeting of the Society for Psychotherapy Research, UK-Chapter; panel presentation

[B24] Hinson VK Weinstein S Bernard B Leurgans SE Goetz CG Single-blind clinical trial of psychotherapy for treatment of psychogenic movement disorders Parkinsonism Relat Disord 2006 12 177 180 10.1016/j.parkreldis.2005.10.006 16364676

[B25] Baldoni F Baldaro B Trombini G Psychotherapeutic perspectives in urethral syndrome Stress Med 1995 11 79 84 10.1002/smi.2460110115

[B26] Abbass AA Campbell S Magee K Tarzwell R Intensive short term dynamic psychotherapy to reduce rates of emergency department return visits for patients with medically unexplained symptoms: preliminary evidence from a pre-post intervention study CJEM 2009 11 529 534 19922712 10.1017/s1481803500011799

[B27] Yalom ID The Theory and Practice of Group Psychotherapy 2005 5 New York: Basic Books

[B28] Sheehan DV Lecrubier Y Sheehan KH Amorim P Janavs J Weiller E Hergueta T Baker R Dunbar GC The Mini-International Neuropsychiatric Interview (M.I.N.I.): the development and validation of a structured diagnostic psychiatric interview for DSM-IV and ICD-10 J Clin Psychiatry 1998 59 22 33 9881538

[B29] First MB Spitzer RL Gibbon M Williams JBW Benjamin L Structured clinical interview for the DSM-IV axis II personality disorders (SCID-II). (Version 2.0) 1994 722 West 168th street, New York, New York: Biometric Department, New York State Psychiatric Institute 10032

[B30] Monsen JT Monsen K Solbakken OA Hansen RS The Affect Consciousness Interview (ACI) and the Affect Consciousness Scales (ACS): Instructions for the interview and rating 2008 Oslo: Department of Psychology, University of Oslo

[B31] Solbakken OA Hansen RS Havik OE Monsen JT The assessment of affect integration: validation of the affect consciousness construct J Pers Assess 2011 93 257 265 10.1080/00223891.2011.558874 21516584

[B32] Lambert MJ Lunnen K Umphress V Hansen N Burlingame GM Administration and scoring manual for the Outcome Questionnaire (OQ-45.1) 1994 Salt Lake City: IHC Center for Behavioral Healthcare Efficacy

[B33] Derogatis LR Rickles K Rock AF The SCL-90 and the MMPI: a step in the validation of a new self-report scale Br J Psychiatry 1976 128 280 289 10.1192/bjp.128.3.280 1252693

[B34] Horowitz LM Alden LE Wiggins JS Pincus AL Inventory of Interpersonal Problems Manual 2000 Odessa, FL: The Psychological Corporation

[B35] Horvath AO Greenberg LS Development and validation of the working alliance inventory J Couns Psychol 1989 36 223 233

[B36] Kadera SW Lambert MJ Andrews AA How much therapy is really enough: a session-by-session analysis of the psychotherapy dose-effect relationship J Psychother Pract Res 1996 5 132 151 22700273 PMC3330412

[B37] Hill CE Lambert MJ Lambert M Methodological issues in studying psychotherapy process and outcomes Bergin & Garfield’s handbook of psychotherapy and behaviour change 2004 New York: John Wiley and sons 84 135

[B38] Tracey JG Rounds J Gurtman M Examination of the general factor with the interpersonal structure: application to the inventory of interpersonal problems Multivar Behav Res 1996 31 441 466 10.1207/s15327906mbr3104_3 26788593

[B39] Monsen JT Hagtvet KA Havik OE Eilertsen DE Circumplex structure and personality disorder correlates of the interpersonal problems model (IIP-C): construct validity and clinical implications Psychol Assess 2006 18 165 173 16768592 10.1037/1040-3590.18.2.165

[B40] Tracey TJ Kokotovic AM Factor structure of the working alliance inventory Psychological Assessment: A Journal of Consulting and Clinical Psychology 1989 1 207 210

[B41] Hox J Multilevel analysis. Techniques and applications 2010 2 New York: Routledge

[B42] Singer JD Willett JB Applied longitudinal data analysis. Modeling change and event occurrence 2003 New York: Oxford University Press

